# Dual drug‐loaded polymeric mixed micelles for ovarian cancer: Approach to enhanced therapeutic efficacy of albendazole and paclitaxel

**DOI:** 10.1111/jcmm.18389

**Published:** 2024-06-12

**Authors:** Nikita Maruti Gaikwad, Pravin Digambar Chaudhari, Karimunnisa Sameer Shaikh, Somdatta Y. Chaudhari, Sandeep S. Pathare, Amir Afzal Shaikh, Nada H. Aljarba, Ajoy Kumer, Bikram Dhara

**Affiliations:** ^1^ Department of Pharmaceutics Modern College of Pharmacy Pune Maharashtra India; ^2^ Department of Pharmaceutical Chemistry Modern College of Pharmacy Pune Maharashtra India; ^3^ Department of Pharmaceutical Chemistry Bharati Vidyapeeth (Deemed to be University), Poona College of Pharmacy Pune Maharashtra India; ^4^ Department of Pharmaceutics SCES's Indira College of Pharmacy “Niramay” Pune Maharashtra India; ^5^ Department of Biology College of Science, Princess Nourah bint Abdulrahman University Riyadh Saudi Arabia; ^6^ Department of Chemistry College of Arts and Sciences, IUBAT‐International University of Business Agriculture and Technology Dhaka Bangladesh; ^7^ Center for Global Health Research Saveetha Medical College and Hospital, Saveetha Institute of Medical and Technical Sciences Chennai India; ^8^ Department of Health Sciences Novel Global Community Educational Foundation Hebersham New South Wales Australia

**Keywords:** albendazole, folic acid, paclitaxel, polymeric mixed micelles, soluplus, TPGS, VEGFR‐2

## Abstract

Chemotherapy resistance remains a significant challenge in treating ovarian cancer effectively. This study addresses this issue by utilizing a dual drug‐loaded nanomicelle system comprising albendazole (ABZ) and paclitaxel (PTX), encapsulated in a novel carrier matrix of D‐tocopheryl polyethylene glycol 1000 succinate vitamin E (TPGS), soluplus and folic acid. Our objective was to develop and optimize this nanoparticulate delivery system using solvent evaporation techniques to enhance the therapeutic efficacy against ovarian cancer. The formulation process involved pre‐formulation, formulation, optimization, and comprehensive characterization of the micelles. Optimization was conducted through a 32 factorial design, focusing on the effects of polymer ratios on particle size, zeta potential, polydispersity index (PDI) and entrapment efficiency (%EE). The optimal formulation demonstrated improved dilution stability, as indicated by a critical micelle concentration (CMC) of 0.0015 mg/mL for the TPGS‐folic acid conjugate (TPGS‐FOL). Extensive characterization included differential scanning calorimetry (DSC), nuclear magnetic resonance (NMR), and Fourier‐transform infrared spectroscopy (FTIR). The release profile exhibited an initial burst followed by sustained release over 90 h. The cytotoxic potential of the formulated micelles was superior to that of the drugs alone, as assessed by MTT assays on SKOV3 ovarian cell lines. Additionally, in vivo studies confirmed the presence of both drugs in plasma and tumour tissues, suggesting effective targeting and penetration. In conclusion, the developed TPGS‐Fol‐based nanomicelles for co‐delivering ABZ and PTX show promising results in overcoming drug resistance, enhancing solubility, sustaining drug release, and improving therapeutic outcomes in ovarian cancer treatment.

## 1 INTRODUCTION

Polymericnanomicelles represent a versatile and promising nano platform for a wide range of biomedical applications. These nanostructures consist of amphiphilic block copolymers self‐assembled into core‐shell nanoparticles, where the hydrophobic segments form the core, while the hydrophilic segments constitute the shell. This unique structure imparts several advantageous properties to polymeric nanomicelles, making them promising candidates for various therapeutic and diagnostic purposes. One of the key benefits of polymeric nanomicelles lies in their ability to solubilize poorly water‐soluble drugs, thereby enhancing their bioavailability and therapeutic efficacy. The hydrophobic core of nanomicelles serves as a reservoir for accommodating hydrophobic drug molecules, protecting them from degradation and facilitating their transport through biological barriers. This property is particularly advantageous for delivering drugs with low aqueous solubility. Their small size allows for passive accumulation in diseased tissues via the enhanced permeability and retention (EPR) effect, wherein nanoparticles preferentially accumulate in tumours due to leaky vasculature and poor lymphatic drainage. This phenomenon can improve drug targeting to specific tissues while minimizing systemic toxicity.^1^

Along with the platinum drugs, paclitaxel is considered a first‐line medication for the chemotherapeutic treatment of ovarian cancer. Paclitaxel, which is derived from the *Taxus brevifolia* tree, which is also known as the Pacific yew, has made considerable strides since the first discovery of its antineoplastic capabilities and has since been used in clinical.^2^ Many advanced nano‐particulate drug delivery systems have been developed for paclitaxel drugs. Paclitaxel has been delivered using a variety of drug delivery techniques, including drug‐polymer conjugation, lipid‐based nanoparticles, polymeric nanoparticles, gold and magnetic nanoparticles. For paclitaxel, several innovative drug carrier systems, including carbon nanotubes, nanocrystals, nanogel and cyclodextrin nanoparticles, have also been built. Now, albumin‐bound paclitaxel nanoparticles are the only formulation approved bythe FDA.^3^ Albendazole (ABZ), is a benzimidazole‐carbamate anti‐anthelmintic medication, with a wide range of benefits in opposition to hydatid cysts. Albendazole primarily works by inhibiting microtubule polymerization by binding with tubulin, acting as an antiparasitic drug, and causing the breakdown of the parasite's microtubule system. Additionally, its interaction causes the cell cycle to stop at the G2 M phase. Albendazole proved to be a fumarate reduction enzyme inhibitor, which many helminths utilize as a respiratory chain. According to reports, albendazole has a strong inhibitory effect on both VEGF and HIF‐1 (hypoxia‐inducible factor 1). The expression of VEGF at the site of the tumour elevated together with an increase in HIF‐1 during tumour progression.^2^ Overexpression of VEGF at tumour sites leads to the formation of new blood vessels called angiogenesis. Tumour development, progression and metastasis are aided by the production of new blood vessels. Recently, ABZ grabbed attention as a potent anticancer agent. Numerous cancer cells, including colon, colorectal, hepatocellular carcinoma and others like lung cancer, and ovarian cancer have been examined about albendazole. Targeted medication distribution is a potential method for improving therapeutic agent effectiveness and safety. This is especially useful for illnesses like cancer, where the medicine's dose‐limiting toxicity and the development of drug resistance are key impediments to therapeutic effectiveness. Among the cellular surface targets that might be used in therapeutic targeting, the folate receptor (FR) is one of the most promising and studied epithelial cancer indicators.^3^, ^4^ The purpose of this research is to arrange nano micellesto facilitate the administration of the combination chemotherapy drugs paclitaxel and albendazole as a treatment for ovarian cancer. Paclitaxel is a well‐known anticancer drug that targets microtubules in the cells. Paclitaxel acts by binding to the beta subunit of tubulin, thereby stabilizing microtubules and preventing their depolymerization. This stabilization leads to the formation of abnormal, non‐functional microtubule bundles within the cell. As previously reported work on the molecular docking study of albendazole on VEGFR‐2 receptor, it strongly blocks the VEGFR signalling responsible for the formation of new blood vessels. So, by blocking the action of VEGFR‐2 it prevents the growth of tumours. Anticancer properties of albendazole are also reported in some research articles. The addition of Vitamin E (TPGS 1000) to naomicelles led to an improvement in their ability to hit their target, which suggests that this therapy method may be superior. TPGS possesses a unique combination of properties that make it a versatile ingredient in pharmaceuticals, cosmetics and food products. Its solubility‐enhancing, emulsifying, antioxidant, biocompatible and drug delivery‐enhancing properties contribute to its widespread use across various industries. TPGS can also modulate drug absorption and permeability across biological membranes. It has been shown to inhibit P‐glycoprotein (P‐gp), a drug efflux transporter involved in multidrug resistance, thereby enhancing the oral bioavailability of certain drugs. Soluplus is a versatile excipient with a unique amphiphilic structure that offers significant advantages in enhancing the solubility, dissolution rate, stability, and tailored release of poorly water‐soluble drugs. Its compatibility with various processing methods and regulatory acceptance makes it an attractive option for formulating pharmaceutical dosage forms with improved therapeutic performance. Folic acid helps to increase the targeting efficiency of nanomicelles. In addition, therapy with albendazole helps overcome paclitaxel resistance in cancer patients.^5^ This study may support for achievement of advanced targeted dual drug delivery with polymeric mixed micelles for ovarian cancer.

## 2 MATERIALS AND METHODS

### 2.1 Materials

Vitamin E (TPGS‐1000) was procured from Connell India & PMC Isochem, Lavoisier, France. Paclitaxel obtained from Cipla Pvt Ltd, Mumbai. Albendazole obtained from sequent pharma, Mumbai. 1,1 carbonyldiimidazole (CDI), dicyclohexylcarbodiimide (DCC), N‐hydroxysuccinamide (NHS), folic acid was purchased from Analab fine chemicals, soluplus was supplied by BASF. Glutaric acid, pyridine, ethylene diamine, DMSO, DMF, acetonitrile, dialysis bags, and the rest of the chemicals and solvents utilized were of analytical grade.

### 2.2 Preparation of TPGS‐Folate conjugation

#### 2.2.1 Activation and synthesis of TPGS‐NH_2_

TPGS first reacts with CDI to generate an intermediary called an imidazole carbamate. In summary, the reaction was conducted at 37°C for 2 h using TPGS 1000 and CDI (molar stoichiometric ratio 1:5) dissolved in dioxane. The final product, the TPGS‐CDI intermediary, was obtained by precipitating the reaction mixture with cold diethyl ether and drying it for 24 h in a vacuum oven.^6^, ^7^

#### 2.2.2 TPGS‐NH_2_ activation

TPGS was activated by NH_2_ by reacting ethylenediamine with an intermediary of TPGS‐CDI to produce amino‐terminated TPGS‐NH_2_. In particular, TPGS‐CDI and ethylenediamine were dissolved at DMSO in a molar ratio of 1:5 and mixed continuously for 24 h in a nitrogen environment at 37°C ± 0.5°C. The mixture was thereafter dialyzed for 48 h first in opposition to DMSO and then for a further 48 h in opposition to extremely pure water. To create TPGS‐NH_2_ powdered form, the residual solution in the dialysis bag was subsequently freeze‐processed.

#### 2.2.3 Activation of TPGS 1000 by folic acid

TPGS activated by folic acid by dissolved TPGS‐NH_2_, folic acid, DCC and NHS (stoichiometric molar ratio 1:1:2:2) in DMSO. Folate activation has been done by the reaction of FOL to DCC and NHS, which engage with the TPGS amino terminus in the carboxylic group to create TPGS‐FOL with water molecules. TPGS was activated by NH_2_ by reacting ethylenediamine with an intermediate of TPGS‐CDI to form amino‐terminated TPGS‐NH_2_. In specifically, TPGS‐CDI and ethylenediamine were dissolved in DMSO in a 1:5 molar ratio and continuously mixed for 24 h in nitrogen surroundings at ambient temperature. The mixture was subsequently dialyzed for 48 h against DMSO and then against ultrapure water. For the production of TPGS‐NH_2_ powdered form, the leftover solution in the dialysis bag was frozen.

### 2.3 Characterization of TPGS 1000‐FOL conjugation

TPGS‐FOL conjugation was checked and confirmed by CMC and ^1^H NMR.^8^

#### 2.3.1 CMC

The CMC of TPGS 1000 succinate was evaluated by employing the UV method. As per the previously reported method, accurately weighed 1 g I2 and 2 g KI were added in 100 mL distilled water to make a standard KI/I_2_ solution. The different concentrations of polymer solution ranging from 0.001% to 0.1% containing exactly 25 μL of iodine solution in each sample were prepared for CMC determination. Before determination all samples were kept in the dark place for 12 h. Using a UV spectrophotometer, the absorbance of various polymer solution concentrations was determined at 366 nm. The CMC value of micelles was calculated by plotting a graph of absorbance versus log of polymer mass concentration.

#### 2.3.2 Nuclear magnetic resonance (NMR)

^1^H NMR spectra of TPGS‐FOL conjugation were performed on Bruker Avance at 400 MHz. The DMSO solvent was used for determination.

### 2.4 Formation and evaluation of micelles preparation

The solvent evaporation method was used to form the polymeric micelles. Albendazole and paclitaxel were first dissolved in DMF solvent. The polymer, TPGS, and soluplus were then dissolved in DMF, and the drug solutions were added to the polymeric solution. This drug‐polymer mixture was placed in a round bottom rotary flask and rotated for 2 h, maintaining temperature at 40°C. After 2 h, the film was kept overnight for a complete evaporation of organic solvent. After that, with 20 mL of water, the film was moistened while being continuously stirred till blue tint micelles were obtained. During this procedure effect of some process parameters we need to consider like stirring time, temperature and stirring speed. These parameters may affect the formation of micelles as well as control the particle size, entrapment efficiency and zeta potential. In the case of folate loaded micelles, TPGS‐Folate was used in TPGS.^8^, ^9^, ^10^

### 2.5 Evaluation of micelles

#### 2.5.1 Micelles size, zeta potential and PDI

Using an SZ‐100 particle size analyser (Horriba), the size, PDI, and zeta potential of polymeric mixed micelles loaded with ABZ and PTX were evaluated. The mixed micelles formulations obtained by the solvent evaporation method were directly analysed for their particle size. Each sample was measured in triplicate.

#### 2.5.2 Compatibility study of drug‐excipients by FTIR (liquid)

FTIR was used for the determination of compatibility between the drug and polymer. Pour just a small amount of the mixture on one of the KBr plates to be precise. To create a uniform, smooth film, put the second plate on the top and give it a quarter‐turn spin. Put the plates in the sample holder and analyse the spectra. If the sample is particularly concentrated, take the plates off and cleanse one side before putting them back together. To avoid contamination of subsequent samples, the KBr plates must be carefully cleaned following this technique. The spectrum of the sample was compared with standard values.

#### 2.5.3 Entrapment efficiency (EE)

To assess the appropriateness of excipients and the technique of manufacture, drug loading capacity and EE% were evaluated. Entrapment of the drug in micelles was assessed by the HPLC method. The micellar formulation was centrifuged at 7000–8000 rpm for 20 min to remove any free drug. Entrapped drugs ABZ and PTX into the TPGS‐soluplus‐Fol micelles pellets were released by dissolving them into ethanol. A 60:40 v/v mixture of water and acetonitrile containing 0.1% TFA and a rate of flow of 1.0 mL/min served as the mobile phase for the measurement of ABZ and PTX. The detection wavelength of the PDI detector was tuned at 230 nm and 10 μL of solutions were infused in triplicate. The drug entrapment efficiency (Equation 1) was calculated as follows:

(1) $$\mathrm{EE}\;\left(℅\right)=\frac{\text{Weight of initial drug}-\text{Weight of free drug}}{\text{Weight of initial drug}}\times 100$$

#### 2.5.4 DSC

The thermal characteristics of APSTM and APSTFM were measured using a DSC (TA equipment Q‐20). Samples (5–10 mg) were placed on a metal tray with a heating rate 10°C/min. The upper temperature was specified at 250°C and the rate of cooling at 5°C/min. As the purge gas, nitrogen gas was chosen, and the rate of flow was specified at 50 mL/min.

#### 2.5.5 Transmission electron microscopy (TEM) assessment

At a 200 kV increasing voltage across it, a transmission electron microscope (TEM) (FEI, Tecnai G2 T20) was used to determine surface properties of folate attached polymeric mixed micelles (APSTFM). According to standard procedure evaluation was done on TEM. A drop of the mixed micelle solution was put onto the sample cavity prior to TEM examination by appropriately holding the O‐ring. The films were dried in air for 8 min before being examined under a TEM.

#### 2.5.6 In‐vitro release study

Evaluation of drug release from formulation was performed using dialysis bag method. For the drug release investigation, recreation of the conditions, pH values of 5.5 and 7.4 were employed at the particular site environment. First, formulation was centrifuged to obtained drug entrapped pellets. The pellets were collected equivalent to amount of drug present in the final formulation (APSTFM). In brief, 7.5 mg of ABZ and 7.5 mg PTX micelles added into the dialysis bag (Mw 12,000 Da) and 0.5% Tween 80 was added into 50 mL of dissolution media (PBS 7.4 and 5.5 pH). The temperature of entire system was kept at 37°C ± 0.5°C with continuous at 100 rpm on magnetic stirrer. To maintain sink conditions, the sample was withdrawn and replaced with new medium at regular intervals. The quantity of ABZ and PTX released from micelles was measured using the HPLC technique described earlier in % entrapment determination.^11^, ^12^

#### 2.5.7 Release kinetics of drug

The drug's release kinetics was examined by PCP Disso v3 software using the model fitting approach. The model with the highest correlation coefficient was chosen as the best fit model for a specific formulation.

#### 2.5.8 Stability study

##### Stability under storage condition

The storage stability of the final optimized formulation APSTFM and APSTM were determined at 4°C for 45 days. Stability of formulation and effect on storage condition on particle size was checked after 45 days.^8^

##### Dilution stability

The dilution effect on micelles stability was investigated using dilution method. The APSTM and APSTFM were diluted with 10, 50 and 100 times with water for injection and formulations were measured for its mean particle size, PDI and zeta potential.

### 2.6 Ex‐vivo cell experiments

#### 2.6.1 MTT assay

For the MTT assay study, SKOV3 cell line was utilized. Live cells break the light yellow substrate referred to as MTT [3‐(4,5‐dimethylthiazol‐2‐yl)‐2,5‐diphenyl tetrazolium bromide] to create the dark blue formazan outcome. Even newly died cells do not cleave a significant amount of MTT, and this action requires the existence of active mitochondria. As a result, colorimetric techniques show that the measure of MTT broken is proportional to the quantity of live cells that exist. To produce a variety of test concentrations, the mixture were immersed in DMSO and successively diluted with the whole medium. In all samples, the DMSO content was fixed at 0.1%. SKOV3 cells were seeded in plates with 96 wells, subjected to varying concentrations of the experimental substances, and then incubated for 96 h at 37°C with 5% CO_2_. Following the application of the MTT solution during a 4‐h incubation period, the dark blue formazan reagent that resulted from the cells was immersed in DMSO and detected at 550 nm in a safety cabinet. Inhibition percentages were calculated and presented in relation to the concentrations required to calculate IC_50_ readings.^13^, ^14^

#### 2.6.2 Experimental dose determination study

BALB/c mice are commonly employed in immunological research, in part because they exhibit TH2‐biased immune responses. BALB/c is an albino, laboratory‐bred house mouse breed from which several common sub‐strains are developed. First, animals were split into three groups each containing four animals. The concentrations of PTX, ABZ, and APSTFM were used in the dosage determination investigation ranged from 100 to 5000 mg/kg. Test animals were treated with formulation to predict survival and mortality rate. For 1 week, the animals were monitored for cage side effects. 1/10 of LD50 dose was selected for experimental dosing.

#### 2.6.3 Hemolytic toxicity

Hemolytic study performed for APSTFM and compared with pure drugs. In brief, 200 μL heparinized blood from BALB/c mice were treated with test formulations (APSTFM, ABZ and PTX) for 30 min at 37°C. After that, blood samples were centrifuged to separate the plasma. Red coloration of the plasma confirms hemolysis.

#### 2.6.4 Antitumour activity

The experiment was carried out on naked mice aged 8–10 weeks. In brief, 1 × 105 murine mammary cancer SKOV cells were infused into the backs of animal and enabled to grow cancer. In experimental animals, tumours were dissected and regrafted. After the tumour had grown to a palpable size, the test sample was administered. The test sample formulation and API are given once every 2 days at a dosage of 10 mg/kg equivalents i.p., whereas the untreated animals were given PBS IP. Using digital vernier callipers from Mitutoyo Japan, the tumour volume was calculated. The animals were sacrificed by cervical dislocation towards the conclusion of the test. Tumours was eliminated when the mice were dissected. The removed tumours were scanned right away.

#### 2.6.5 Pharmacokinetics study (single point)

Pharmacokinetic study was performed to detect the concentration of drug in the plasma at single point. Test formulation dose of 100 mg/kg was given to the BALB/c by intra‐peritoneal route. After 3 h, blood samples were taken through orbital puncture and plasma was separated using centrifugation techniques. After that the plasma was precipitated with methanol 1:1 and concentration of both drug ABZ and PTX in the plasma was quantify using HPLC method.

#### 2.6.6 Tumour bio‐distribution studies

Tumour bio‐distribution study was performed to detect the concentration of drug reached into tumour at single point. Test formulation dose of 100 mg/kg was given to the BALB/c by cervical dislocation. After 3 h, tumour sample were harvested and stored at freezing temperature. Then tumour samples were homogenates and 500 mg/mL tissue sample were prepared. After homogenization supernatant were extracted and mixed with methanol and then evaluated by using HPLC technique.

## 3 RESULTS

The benefits of medication repurposing in terms of time and cost; make it an excellent alternative method to the standard technique of anticancer drug research and development. Albendazole is a benzimidazole carbamate and primarily an anti‐helmintic medicine with a long history of effective and consistent use in both humans and animals to eradicate parasitic worms, but recently it is repurposed as anti‐cancer drug. ABZ acting on VEGFR‐2 and prevent the angiogenesis. Angiogenesis is the development of new blood vessels which responsible for tumour growth. Paclitaxel is well‐known anti‐cancer drug that used in various cancer conditions. Albedazole activity in paclitaxel resistance cells also reported. Its limited water solubility and cytotoxicity are still regarded as major challenges in developing formulations for therapy effectiveness. One significant function of Vitamin E TPGS is to improve antitumour effectiveness. It also inhibits drug efflux by blocking the P‐gp channel. FRs are overexpressed in ovarian cancer patients. Folic acid can therefore boost the targeted efficacy of micelles.

### 3.1 Preparation of polymeric mixed micelles

#### 3.1.1 Conjugation reaction of TPGS‐Folate

**FIGURE 1:**
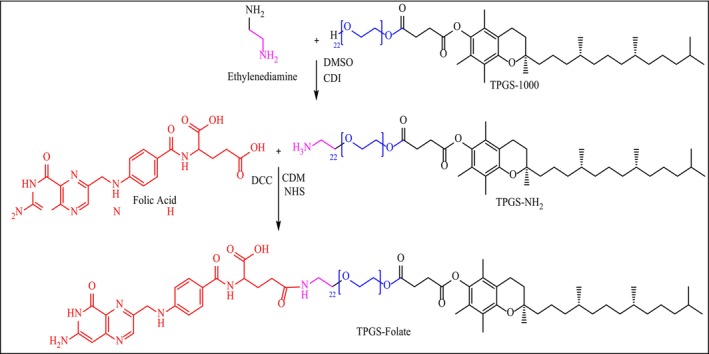
Schematic diagram of TPGS‐Fol conjugation reaction.

Figure 1 showed schematic diagram of TPGS‐Fol conjugation reaction.

#### 3.1.2 Characterization of TPGS‐FOL conjugation

##### CMC

**FIGURE 2:**
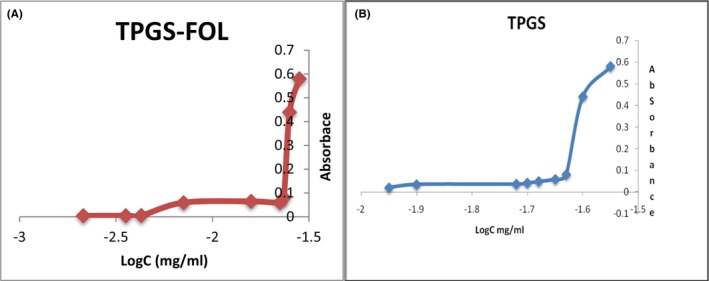
(A) CMC graph of TPGS‐FOL (B) CMC graph of TPGS.

The CMC value of TPGS‐FOL was estimated to be 0.0150 mg/mL (shown in Figure 2A), which was significantly lesser than TPGS (0.0205 mg/mL) (Figure 2B). The decline in CMC showed that the TPGS 1000‐FOL mixed micelles would give excellent drug stability in solution as well as high resistance to breakdown even when diluted by the body's much larger volume of blood. TPGS 1000‐FOL substance are efficiently distributed in the aqueous solution at low concentrations. The free energy of the system increases as the concentration increases due to conversely, a negative relationship within the lipophilic domain and the neighbour water molecule. At a determined concentration, the hydrophobic segments are separated from the water‐based environment by the CMC, an amphiphilic material with the necessary geometrical positioning resulting in the creation of colloidal assemblies known as micelles.

##### Nuclear magnetic resonance (NMR)

**FIGURE 3:**
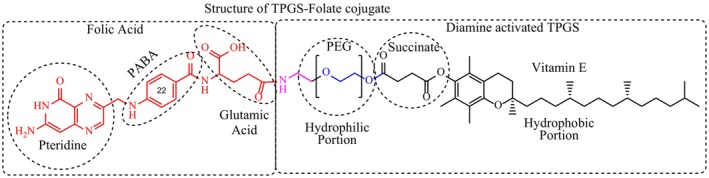
Structure of TPGS‐Fol conjugation.

**FIGURE 4:**
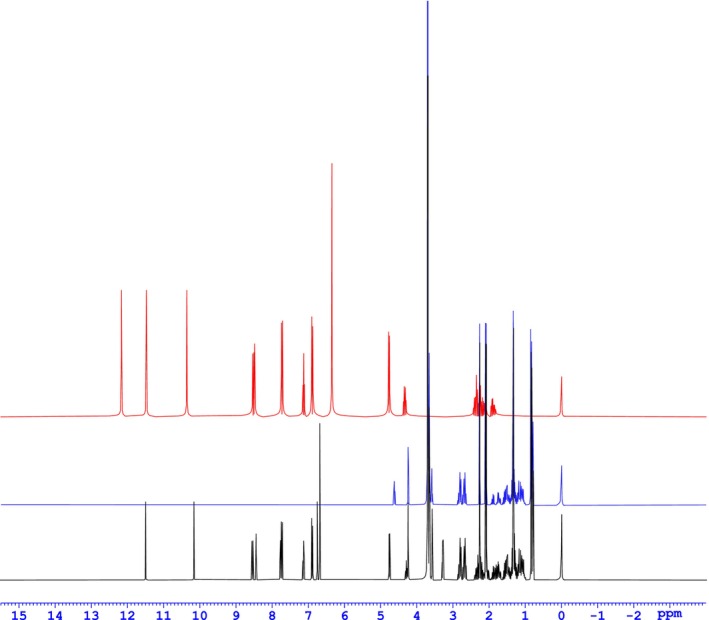
Overlapped ^1^HNMR graphs of folic acid (red), TPGS (blue), TPGS‐Folate (black).

Observations of ^1^H NMR spectroscopy were made to determine the molecular conjugation of folate on TPGS 1000 (Figure 3). Overlapped ^1^HNMR spectrums of folic Acid (red), TPGS 1000 (blue), and TPGS 1000‐Folate conjugation are shown in Figure 4. It clearly indicates appropriate changes in structure of compounds after conjugation. The typical peaks of all the functions present in the structure are mentioned in the Table 1.

**TABLE 1:** Details of NMR spectrum of TPGS‐Folate.

Type of function		Functional group	Peak (δ in ppm)	Signal
Folic acid	Pteridine	—NH_2_	6.680	m
		—NH—	10.150	s
		C—H (pyrimidine)	6.748	m
		C‐H (piperazine)	8.435	m
	PABA	Ar—NH—	7.125	t
		Aromatic C—H	6.889–7.725	m
	Glutamic acid	—NH— (attached to PABA)	8.537	d
		—OH	11.483	s
		3^0^—C—H	4.277	m
TPGS	PEG	C—H (attached to succinate)	4.243	m
		All —CH_2_—	3.6–3.7	m
	Succinate	All —CH_2_—	2.670–2.808	m
	Vitamin‐E	All —CH— and —CH_2_— (alicyclic)	1.000–2.815	m
		All —CH_3_	0.852	m

*Note*: m: mortality and s: survival or morbidity in 50% animals. *n* = 4.

The table indicated all the functional protons available in the TPGS‐Folate conjugate. The spectra of TPGS 1000‐Folate depicts usual folic acid peaks like free —NH_2_ of pteridine ring at 6.680 ppm, ring —NH— at 10.150 ppm, aromatic C—H of para‐amino benzanilide in between 6.889–7725 ppm, O—H of glutamic acid at 11.483 ppm as a singlet, N‐H of Glutamate at 8.537 ppm as a doublet. The spectra of the TPGS 1000‐Folate is very fierce peak at 3.698 ppm that belongs to methylene proton (—CH_2_—) of the PEG (hydrophilic portion) in TPGS. As shown in the overlapped spectra of TPGS 1000‐Folate consists of all characteristic signals beginning with folic acid and TPGS 1000, except the peak of —OH present in folic acid at 12.160 ppm. ^1^HNMR peak intensity of folic acid (441 g/mol) is relatively higher than that of TPGS‐Folate because of low molar weight of folic acid as contrast with TPGS and TPGS‐Folate (1965 g/mol). All ^1^HNMR show the exact number of hydrogens signals that are present in the corresponding chemical structure. This indicates that the conjugation reaction is done properly.

### 3.2 Formulation of polymeric mixed micelles

Polymeric nanoparticles were often produced using the solvent evaporation approach. Solvent evaporation is the process of emulsifying polymer in aqueous phase and dispersing it in a volatile solvent such as CH_2_Cl_2_, CHCl_3_ or ethyl acetate. The polymer components were first dissolved in volatile organic solvents in 3:2 ratio (TPGS: soluplus), and drug in the ratio of (1:1) then the emulsion was produced by solvent evaporation technique. High temperatures, vacuum, or constant stirring are used to evaporate the solvent. The size of the particles may be regulated by modifying factors such as evaporation temperature, evaporation rate, stirring rate and so on. Following the evaporation of the solvent from the polymer solutions, the emulsion is eventually transformed into a polymeric nanoparticle suspension (shown in flowchart).

**Figure:**
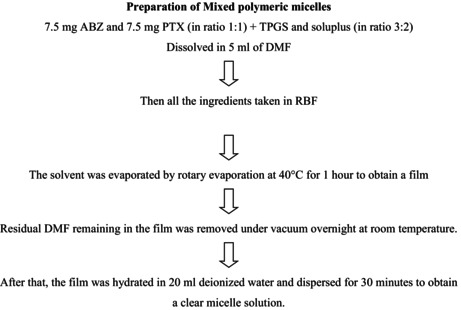
Flow chart of polymeric micelles preparation.

### 3.1 Evaluation of micelles

#### 3.1.1 Mean particle size, zeta potential and PDI analysis

**FIGURE 5:**
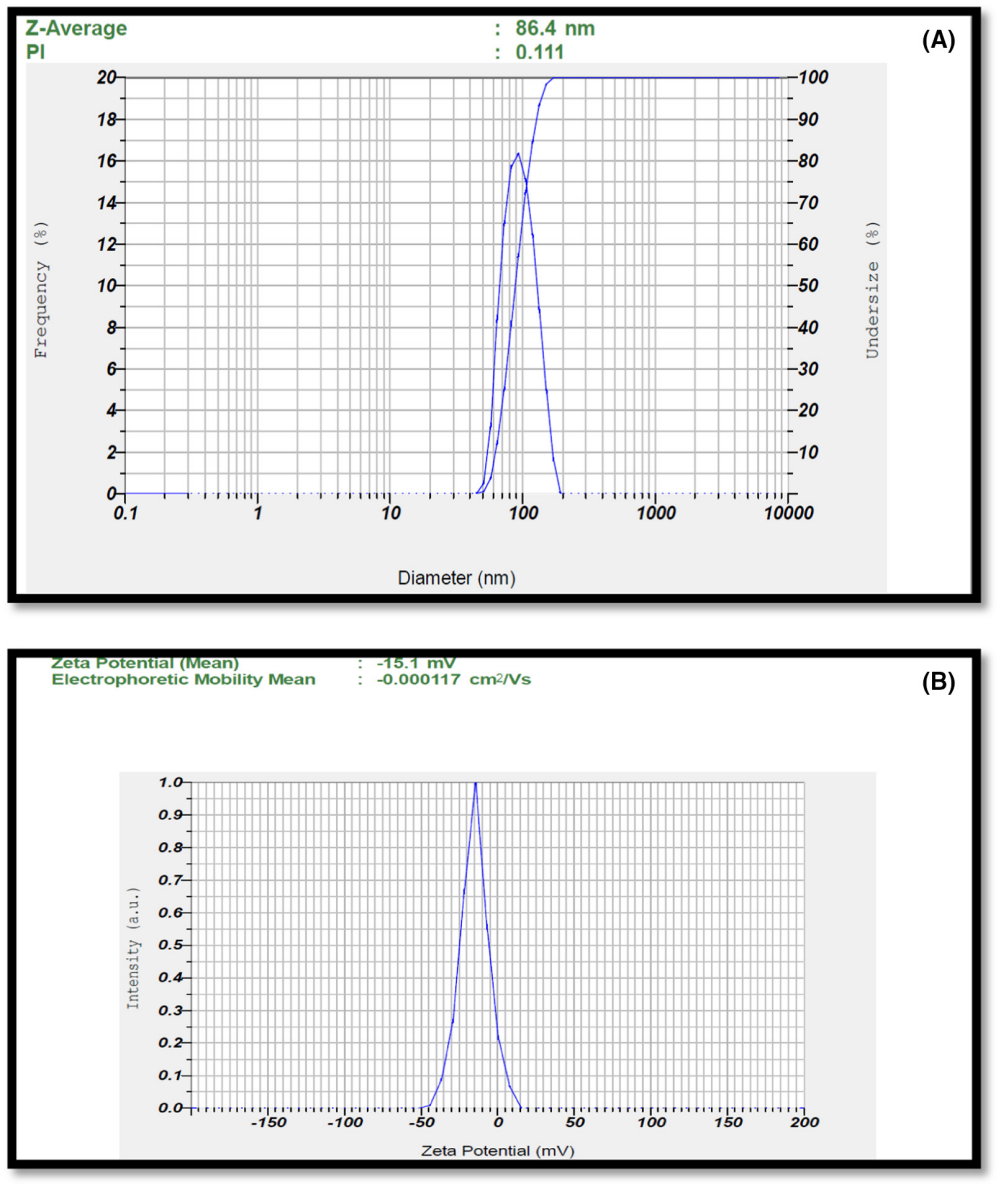
(A) Particle size distribution of APSTM. (B) Zeta potential of APSTM.

**FIGURE 6:**
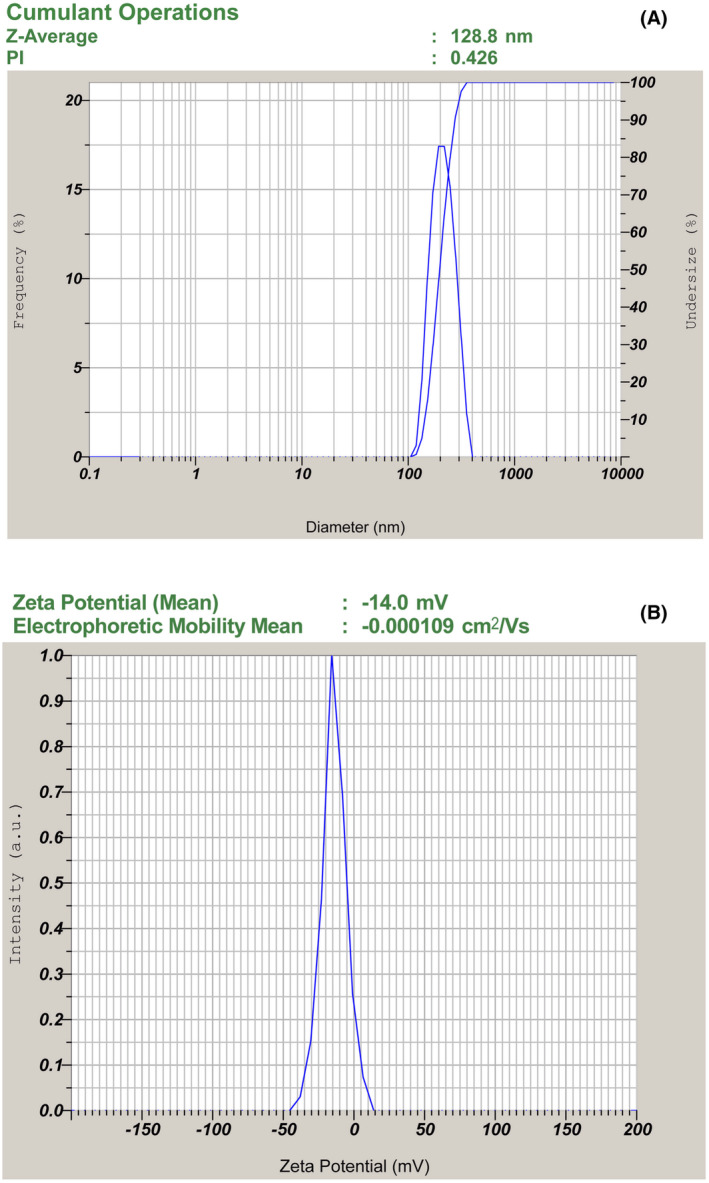
(A) Particle size distribution of APSTFM. (B) Zeta potential of APSTFM.

The size and size distribution of the micelles of optimized formulation APSTM and APSTFM were measured by nano partical Analyser SZ‐100 instrument. The micellar mean size and zeta potential of of APSTM was found to be 87.56 ± 1.25 nm and − 15.68 ± 0.88 mV (Shown in Figure 5A,B) although the average size and zeta potential of APSTFM was determined to be elevated to 128.23 ± 2.49 nm and zeta potential −14.2 ± 0.60 respectively because of folic acid conjugation (shown in Figure 6A,B).

The increased size of APSTFM could be attributed to folic acid conjugation, which promotes micelle expansion. Hydrophilic carboxyl and amino acids, which are capable of forming hydrogen bonds, make up folic acid. A lipophillic drug can be chemically and physically entrapped in polymeric micelles. Chemical conjugation is a chemical process in which physical entrapment results from the hydrophobic interaction of micelles core with drug substances via Van Der Waals forces and hydrogen bonds. Micelles in both the APSTM and APSTFM formulations range in size from 80 to 150 nm, making them appropriate for parenteral drug delivery. Nanoparticles smaller in size (200 nm) can evade phagocytosis by macrophages, even have longer systemic circulation *t* ^1/2^, and are more efficient in target specific action on cancer cells via EPR effect. This is a major benefit since nanoparticles larger over 10 nm may skip clearance through the kidneys.

#### 3.1.2 Compatibility study of drug‐excipients (FTIR)

**FIGURE 7:**
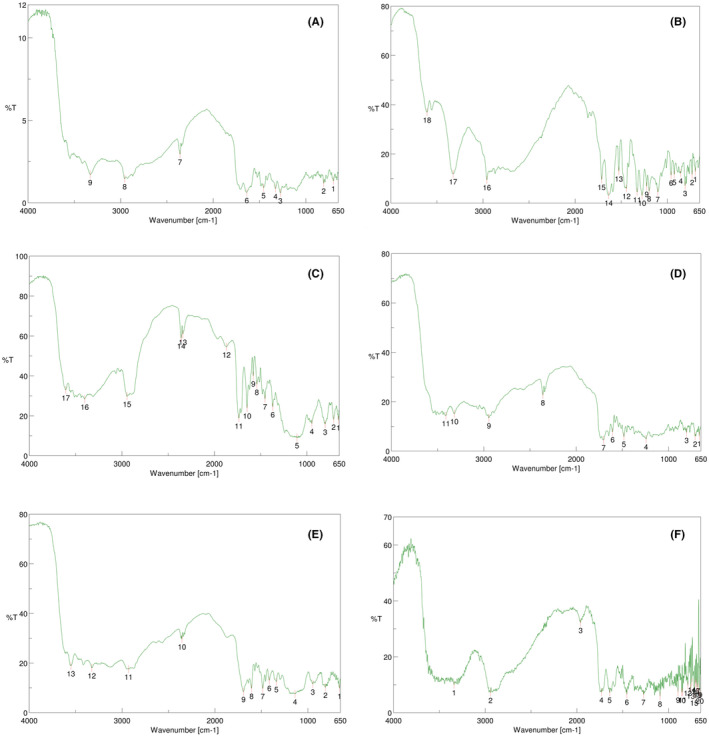
(A) FTIR spectra of ABZ‐PTX‐TPGS‐FA. (B) FTIR spectra of TPGS‐ABZ. (C) FTIR spectra of TPGS‐PTX. (D) FTIR spectra of TPGS‐PTX‐FA. (E) FTIR spectra of TPGS‐FA. (F) FTIR spectra of physical mixture.

FTIR spectra of physical mixture of ABZ, PTZ, Soluplus, TPGS and folic acid were studied for compatibility. The FTIR graph was shown in the Figure 7A–F. The spectral information of IR ranges given in the Tables 2, 3, 4, 5, 6, 7. According to the graphs and observed spectral ranges, both drugs ABZ and PTX are compatible with all three excipients, namely TPGS, Soluplus and folic acid.

**TABLE 2:** FTIR spectral analysis of ABZ‐PTX‐TPGS‐FA.

Characteristics peak	Observed spectra (cm^−1^)	Reported range (cm^−1^)
N—H stretching in carbamate	3328.71	3350–3310
Stretching vibrations of N—H and CH_2_ both symmetric and asymmetrical vibrations	2956.34	3000–2840
N=C=O stretching	2358.52	2250–2349
Bending vibration of C=O bond in carbamate and amide bond	1643.05	1690–1640
—C—C— stretching in aromatic ring	1456.96	1465
—CH_2_ group of PEG chain	1327.75	1350–1300
C—O stretching ester	1274.72	1210–1163

**TABLE 3:** FTIR spectral analysis of TPGS‐ABZ.

Characteristics peak	Observed spectra (cm^−1^)	Reported range (cm^−1^)
N—H stretching (alcohol)	3607.2	3700–3584
N—H stretching	3324.68	3350–3310
Stretching vibrations of N—H and CH2 both symmetric and asymmetrical vibrations.	2958.27	3000–2840
Carbonyl band (—C=O) aldehyde	1713.44	1740–1720
C=O carboxylic group, C=N bending stretching primary amide	1636.3	1600–1670
C—H bending alkane	1443.46	1450–1465
C=C stretching	1606	1620–1610
—CH_2_ group of PEG chain	1327.75m^−1^	1350–1300
C—O stretching ester	1273.75	1210–1163

**TABLE 4:** FTIR spectral analysis of TPGS‐PTX.

Characteristics peak	Observed spectra (cm^−1^)	Reported range (cm^−1^)
N—H stretching (alcohol)	3607.2	3700–3584
N—H stretching vibrations	3402.78	3500–3400
CH2 both symmetric and asymmetrical vibrations and C=O stretching vibration from the ester groups	2944.77	3000–2840
N=C=O stretching	2358.58–2342.12	2250–2349
C=O stretching (anhydride)	1868.68	1540–1870
Carbonyl band (—C=O) aldehyde	1735.62	1740–1720
Amide bond	1645.95	1690–1640
N—O stretching nitro compound	1540.85	1550–1500
C—N and C—O stretching vibration	1452.14	1440–1395

#### 3.1.3 Entrapment efficiency (EE)

The drug entrapment into both micelles formulation APSTM &APSTFM were detected using HPLC. The drug entrapment of ABZ and PTX in APSTM was found to be 67.32% ±2.22 and 78.33% ± 1.45, respectively. The encapsulation of ABZ and PTX in APSTFM was found to be 57.62% ± 2.48 and 74.96% ±0.89, respectively. APSTM showed more percentage of drug encapsulation due to bulky hydrophobic core of TPGS which allow more drugs to entrap.

#### 3.1.4 DSC (differential scanning calorimetry)

**FIGURE 8:**
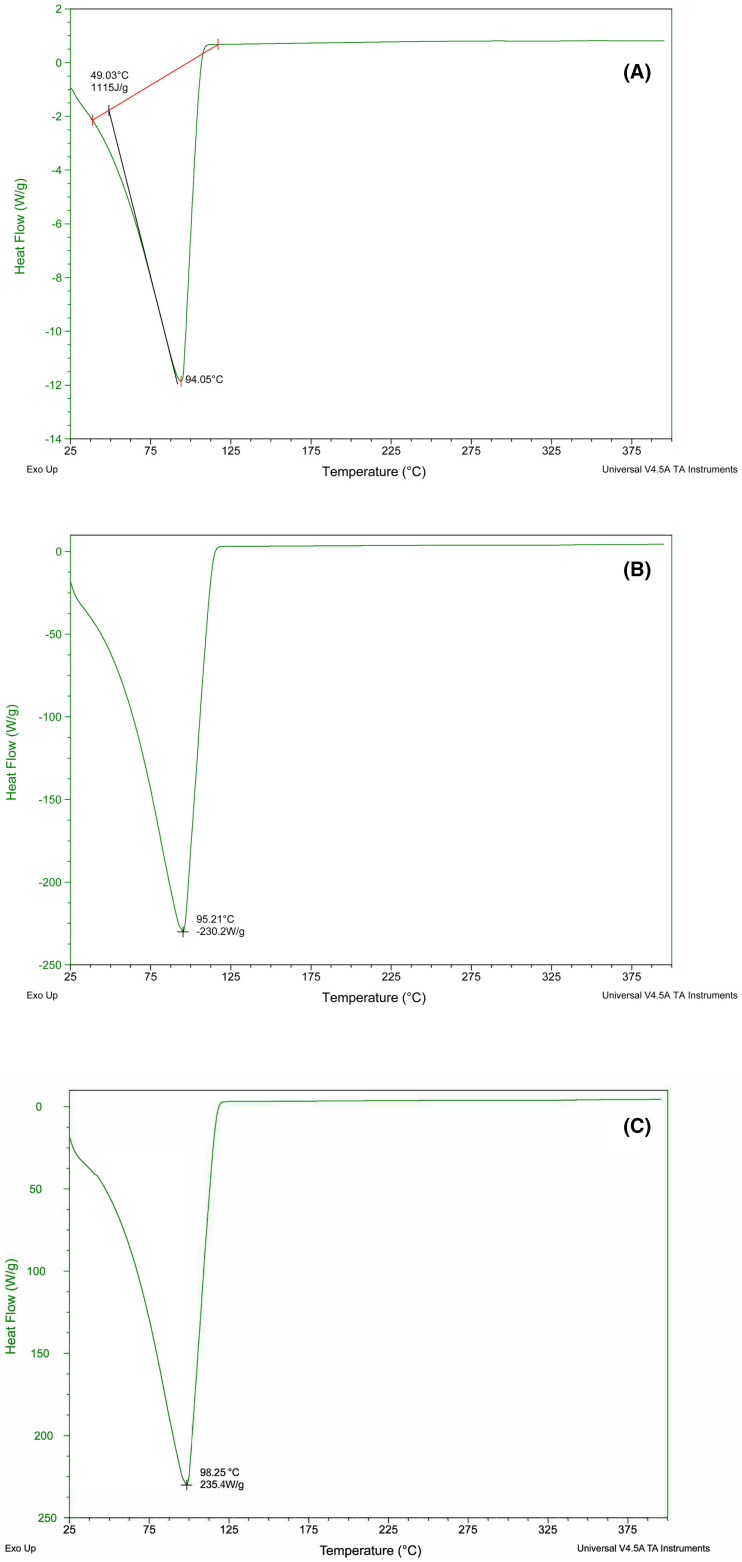
(A) DSC thermogram of Blank micelles. (B) DSC thermogram of APSTM. (C) DSC thermogram of APSTFM.

The free ABZ and PTX peak disappeared in APSTM and APSTFM thermograms, demonstrating molecular dispersion of both compounds in micellar core, where drug‐polymer bonding also affects crystallanity. Figure 8A shows transition of blank micelles at 94.05°C with ΔH 1115 J/g. Figure 8B indicates sharp melting transition of APSTM optimized micelles at 95.21°C with ΔH −230.2 W/g. Figure 8C indicates sharp melting transition of APSTFM optimized micelles at 98.25°C with ΔH −235.4 W/g. The shifting of DSC thermogram from 95.21°C to 98.25°C may be due to increase its molecular weight because of folic acid attached.

**TABLE 5:** FTIR spectral analysis of TPGS‐PTX + FA.

Characteristics peak	Observed spectra (cm^−1^)	Reported range (cm^−1^)
N—H stretching vibrations	3402.78	3500–3400
N—H stretching	3322.75–3418.21	3400–3300
Stretching vibrations of N‐H and CH2 both symmetric and asymmetrical vibrations.	2944.77	3000–2840
N=C=O stretching	2360.44	2250–2349
Carbonyl band (—C=O) aldehyde	1703.8	1740–1720
C=O carboxylic group, C=N bending stretching primary amide	1606.41	1600–1670
—C—C— stretching in aromatic ring	1483.96	1465
C—N stretching vibrations	1243.86	1250–1020

**TABLE 6:** FTIR spectral analysis of TPGS‐FA.

Characteristics peak	Observed spectra (cm^−1^)	Reported range (cm^−1^)
O—H stretching (alcohol)	3551.27	3584–3700
N—H stretching in carbamate	3326.61	3350–3310
Stretching vibrations of N—H and CH_2_ both symmetric and asymmetrical vibrations.	2931.27	3000–2840
N=C=O stretching	2355.62	2250–2349
Bending vibration of C=O bond in carbamate and amide bond	1693.19	1690–1640
—C—C— stretching in aromatic ring	1483.96	1465
—CH_2_ group of PEG chain	1327.75	1350–1300

**TABLE 7:** FTIR spectral analysis of physical mixture.

Characteristics peak	Observed spectra (cm^−1^)	Reported range (cm^−1^)
N—H stretching in carbamate	3340.71	3350–3310
Bending vibration of C=O bond in carbamate and C=O stretching vibration from the ester groups	1732.73	1720–1706
Stretching vibrations of N—H and CH_2_ both symmetric and asymmetrical vibrations	2942.84	3000–2840
C=C=C stretching	1960.29	2000–1900
Bending vibration of C=O bond in carbamate and amide bond	1643.05	1690–1640
C—N stretching vibrations	1094	1250–1020
CH and NH in‐plane bending vibrations	1274	1220–960
—C—C— stretching in aromatic ring	1455.99	1465

**TABLE 8:** Dilution stability of APSTM and APSTFM.

Name of sample	Particle size (nm)		
	Dilution volume, 5 mL	Dilution volume, 10 mL	Dilution volume, 100 mL
APSTM	86.23 ± 2.02	92.9 ± 2.5	95.56 ± 3.01
APSTFM	128.56 ± 1.96	135.56 ± 2.85	140.33 ± 1.52

*Notes:* Values represented in mean±SD. This study examines the effect of dilution on particle size, specifically analyzing changes after diluting by 5, 10, and 100 times. The findings of this study provide insights into the stability of micelles under varying dilution conditions. So, No significant changes observed in particle size after dilution.

**TABLE 9:** Storage stability at 4°C of APSTM and APSTFM.

Time (day)	Particle size (nm)	Particle size (nm)
	APSTM	APSTFM
	(4°C)	(4°C)
5	84.7 ± 2.11	125.86 ± 2.9
10	85.3 ± 1.95	127.16 ± 3.15
15	84.63 ± 2.08	127.6 ± 2.05
20	88 ± 2.52	126.5 ± 2.5
25	85.43 ± 2.09	125.96 ± 3.0
30	83.8 ± 2.50	128.8 ± 2.30
35	88.86 ± 1.52	126.9 ± 2.59
40	86.26 ± 1.61	127.96 ± 2.58
45	92.03 ± 2.56	128.7 ± 2.13

*Notes:* Values represented in mean±SD *n*=3. Studying the effect of temperature on the particle size of micelles at 40°C through storage stability studies is critical for ensuring product stability, optimizing storage conditions, regulatory compliance, and maintaining quality control. The table shows no significant difference in particle size after storage at 40°C, even after 45 days.

**TABLE 10:** MTT assay.

Conc. (μg/mL)	PTX	ABZ	TPGS	APSTM	APSTFM
10	74.51 ± 0.211	34.15 ± 0.153	18.57 ± 0.101	62.38 ± 0.100	88.46 ± 0.064
1	53.26 ± 0.280	18.71 ± 0.153	16.32 ± 0.100	52.62 ± 0.076	56.63 ± 0.090
0.1	28.14 ± 0.300	12.35 ± 0.115	12.58 ± 0.115	41.27 ± 0.100	47.25 ± 0.058
0.01	25.42 ± 0.289	6.69 ± 0.115	6.35 ± 0.058	7.24 ± 0.058	18.19 ± 0.051
0.001	18.26 ± 0.173	3.37 ± 0.115	3.84 ± 0.058	3.54 ± 0.072	2.47 ± 0.049
IC_50_ values	0.7	2	10	0.4	0.2

**TABLE 11:** Dose determination study (LD50).

Dose	PTX	ABZ	APSTFM
100 mg/kg	s	s	s
1000 mg/kg	m	m	m
5000 mg/kg	m	m	m

*Note*: m: mortality and s: survival or morbidity in 50% animals. *n* = 4.

**TABLE 12:** Hemolytic toxicity study.

Dose	PTX	ABZ	APSTFM
10 μg/mL	−ve	−ve	−ve
100 μg/mL	−ve	−ve	−ve
1000 μg/mL	+ve	−ve	+ve

#### 3.1.5 TEM

**FIGURE 9:**
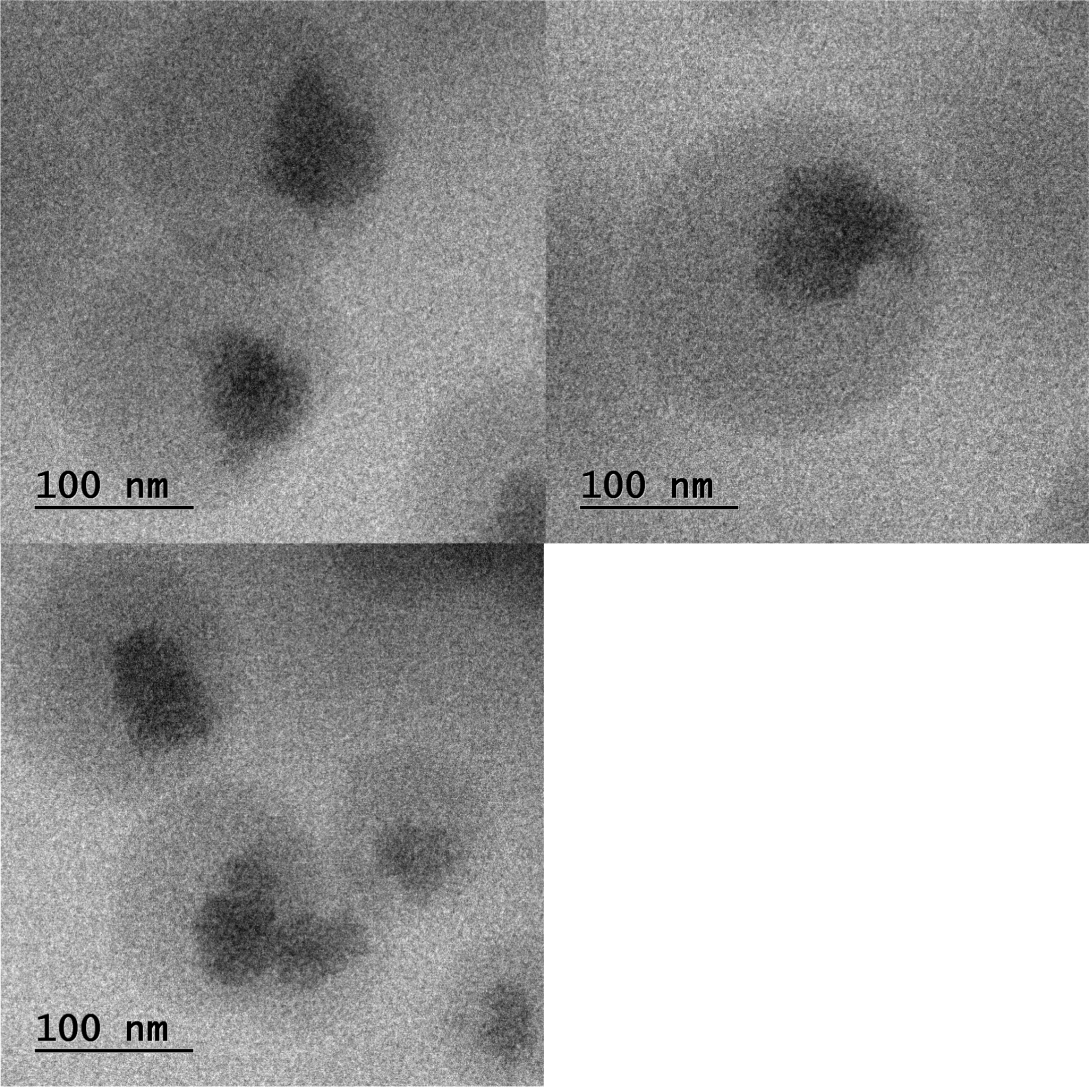
TEM of APSTFM.

The study of surface morphology of final optimized formulation APSTFM was performed by using TEM. The morphology of the micelles from TEM images observed spherical shape, uniform and smooth surface area (shown in Figure 9).

#### 3.1.6 In‐vitro release study

**FIGURE 10:**
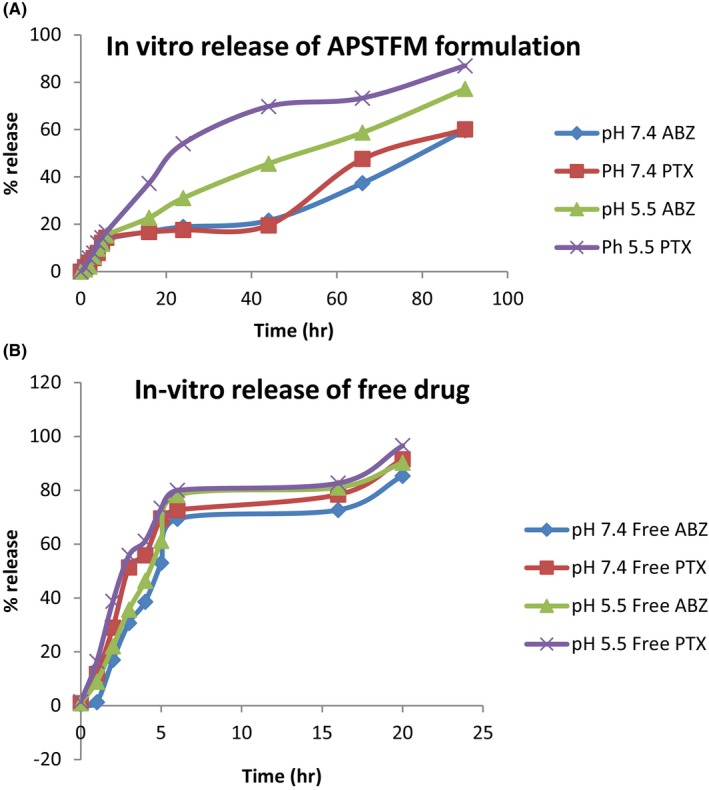
(A) In‐vitro release of drug from APSTFM. (B) In‐vitro release of free drug.

In‐vitro medication releases of ABZ and PTX from polymeric micelles were studied by diffusion bag technique. These micelles formulation meant for systemic administration, both drugs releases were performed in PBS pH 7.4 and though target is ovarian cancer release also checked in slightly acidic environment at pH 5.5 to mimic condition of cells. Figure 10 shows release pattern of both drug from micelles in pH 5.5 and pH 7.4. However, ABZ and PTX release from APSTFM showed biphasic release pattern with initial burst release. The maximum free ABZ and free PTX were released within 16 h in pH 5.5 and pH 7.4 (shown in Figure 10B). So, as compared to pure drug, APSTFM showed sustained release pattern up to 90 h.

#### 3.1.7 Release kinetics of drug

The PCP disso software was used to study release kinetics of optimized formulation. Different t90 values were observed for each drug in different pH. A t90 value indicates 90% drug release at particular duration. Here, ABZ and PTX release from APSTFM were studied in the pH 7.4 mimics the blood pH and pH 5.5 mimic's slightly acidic environment around the tumour cells. In APSTFM, t90 value of ABZ in pH 7.4 was found to be 5529.6 min or 92.6 h or 3.85 days and PTX in 7.4 was found to be 7259.8 min or 120 h or 5 days. For APSTFM, t90 value of ABZ in pH 5.5 was found to be 5042.3 min or 84 h or 3.5 days and PTX in 7.4 was found to be 4911.1 min or 81 h or 3.4 days. The optimized batch APSTFM followed Korsemeyer–Peppas kinetics.

#### 3.1.8 Stability study

##### Dilution stability

The introduction of drug‐encapsulated micelles into the blood, they dilute numerous times and dissociate into monomers. As shown in Table 8, the size of the micelles did not appreciably alter after being diluted in water 5, 10 or 100 times. These findings revealed that APSTM and APSTFM were more resilient to dilution after injections.

##### Storage stability

Micelle size, zeta potential and PDI were used to determine the physicochemical stabilities of APSTM and APSTFM. Both micelles formulations were subjected for 45 days stability study at 4°C. The observation of this study revealed that micelles showed better stability under 4°C temperature as compared to 25°C room temperature because after 5 days only it showed maximum particle size due to aggregation. Hence, stability study was carried out only at 40°C (shown in Table 9). So the storage condition of micelles should be at 4°C.

### 3.2 Ex‐vivo cell experiments

#### 3.2.1 Cytotoxicity study (MTT assay)

**FIGURE 11:**
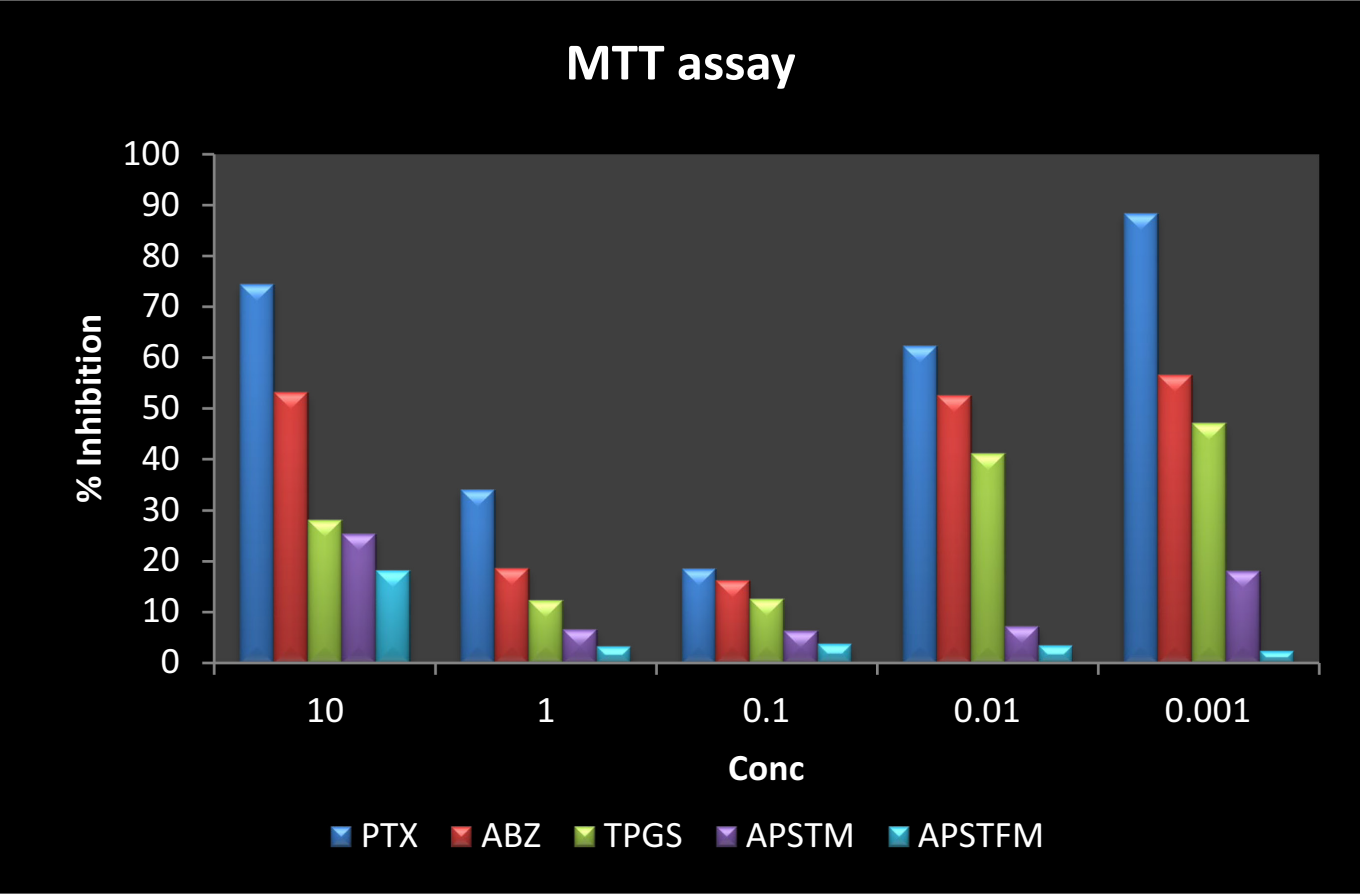
Individual MTT assay graph of samples.

Both formulations demonstrated effective cell inhibition ability in MTT assays when compared to a single drug. MTT assay was employed to evaluate the viability of SKOV3 ovarian cancer cells treated with free ABZ, free PTX, free TPGS, micelles without folate (APSTM) and micelles with folate (APSTFM) as shown in Table 10. The effect was studied using different concentrations of sample ranging from 0.001 to 10 μg/mL. After 96 h of incubation, APSTFM polymeric mixed micelles exhibited considerably better cytotoxicity as compared to other groups. The results demonstrated that IC_50_ of APSTFM (0.2 ± 0.039 μg/mL) much lower than free drugs ABZ (2 ± 0.015 μg/mL) and PTX (0.7 ± 0.039 μg/mL) (shown in Figure 11). The APSTFM polymeric mixed micelles showed higher cytotoxicity than both free drugs may be due to dual drug loaded action. As the polymeric mixed micelles contains TPGS which may help in the internalization of more drugs inside the cell by blocking the action of P‐gp channel. Furthermore, because mixed micelles were encased in endosomes after their entry into the cell, they avoided P‐gp‐mediated efflux, resulting in high intracellular drug concentrations.

#### 3.2.2 Dose determination study

The data from the dose determination study was used to determining the lethal dose. The paclitaxel, albendazole, and APSTFM doses up to 100 mg/kg resulted in 50% animal survival. However, when the dose was increased to 5000 mg/kg, 50% of the animals died. According to the findings, the dose should be kept to a minimum level, that is, 1/10th of the survival dose. Hence, 10 mg/kg was chosen as the final dose for further research (shown in Table 11).

#### 3.2.3 Hemolytic toxicity

Hemolytic toxicity is important when drug parentrally administred. For hemolytic toxicity study 10, 100 and 1000 μg/mL dose was taken. APSTFM and Paclitaxel were showed positive effect at concentration 1000 μg/mL. Paclitaxel already proven as potent drug for cancer treatment. Hence, may be due to that reason at 1000 μg/mL concentration it showed hemolysis. Albendazole does not show any hemolysis at 1000 μg/mL concentration. APSTFM indicated high biocompatibility in the blood at 100 μg/mL dose (shown in Table 12).

#### 3.2.4 Antitumour activity

**FIGURE 12:**
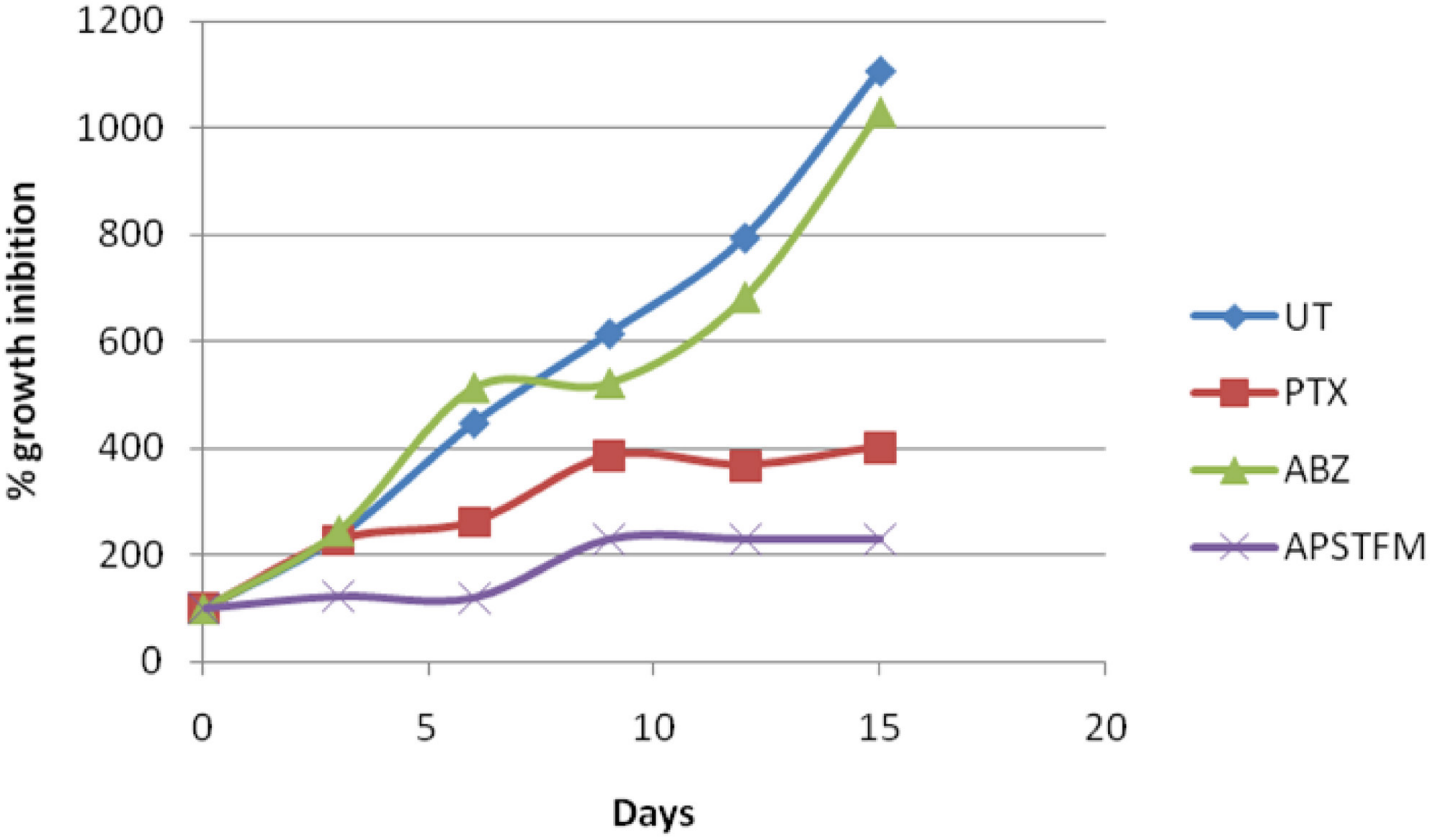
Antitumour activity.

**FIGURE 13:**
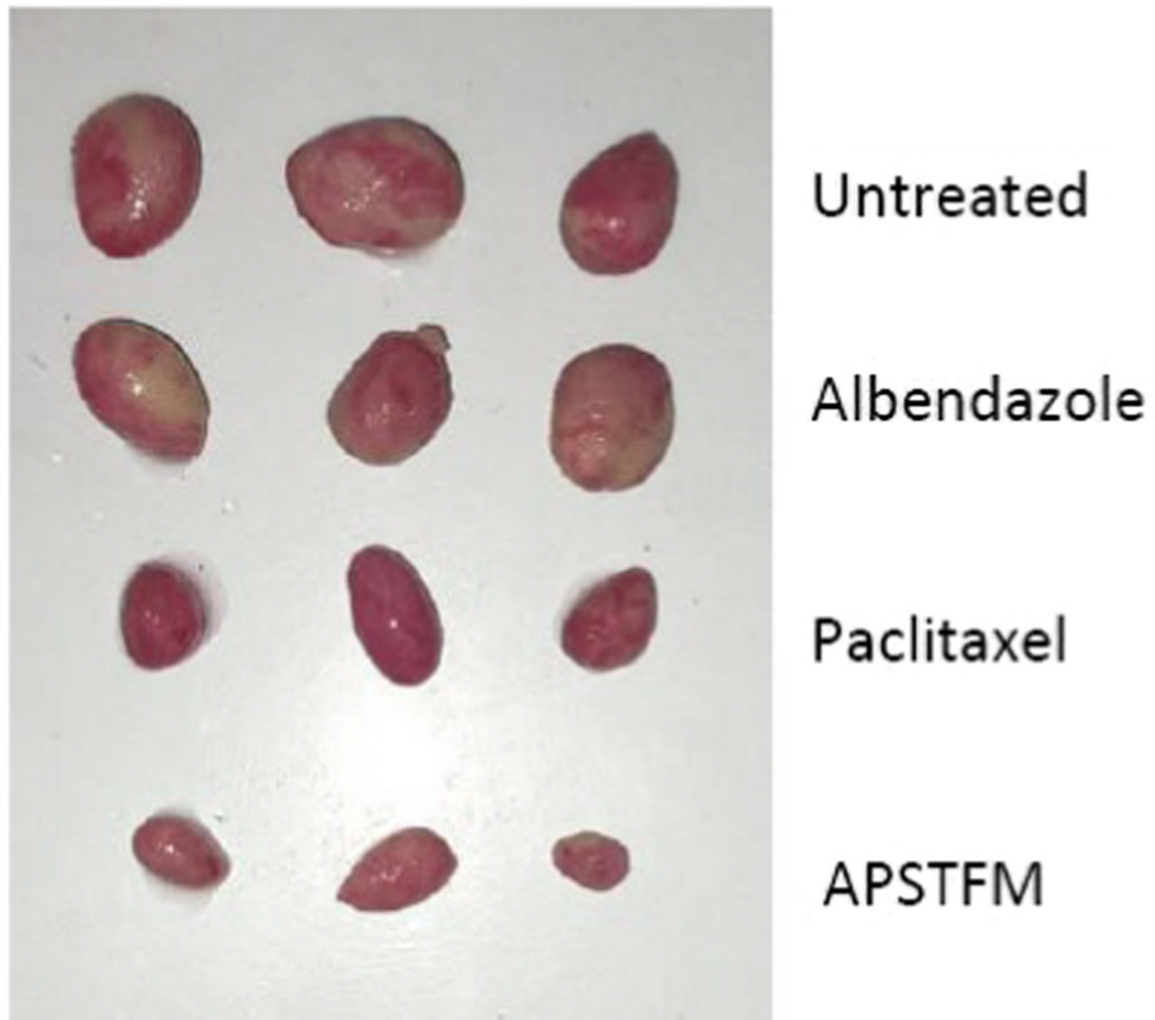
Excise images of tumour after 15 days.

The antitumour potential of APSTFM polymeric mixed micelles was assessed by determining the tumour volume when reached about 100–200 mm^3^. The size of tumours and the body mass of mice were monitored during the 15 days of the treatment. As compared to pure ABZ and PTX, APSTFM formulation more effectively inhibits the growth of tumour.On day 15, the average volume of tumour in the APSTFM was 230.9 ± 96.9 mm^3^ versus 1107.4 ± 110.0 mm^3^ in un‐treated group. As compared to untreated group albendazole not showed significant effect up to 6 days but it showed significant tumour volume reduction after 6–12 days. As paclitaxel is an anticancer drug, it exhibited good antitumour activity and as per the data obtained it also showed significant effect on inhibits the tumour growth. The final nanomicelles formulation (APSTFM) is significantly preventing tumour growth as compared to free albnedazole and free paclitaxel. Hence it is proved that paclitaxel in the combination of albendazole showed more synergistic action on prevent tumour growth. Also efficacy of APSTFM enhanced due to TPGS‐FOL combination. Moreover, the treatment with APSTFM strongly inhibited the growth of tumour during 15 days treatment (shown in Figures 12 and 13).The research protocol outlined herein has been reviewed and approved by the Institutional Animal Ethics Committee, ensuring compliance with all relevant ethical guidelines and standards (CPCSEA letter no. 1582/PO/Re/S/11/CPCSEA).

#### 3.2.5 Pharmacokinetics study plasma distribution and tumour bio‐distribution

**FIGURE 14:**
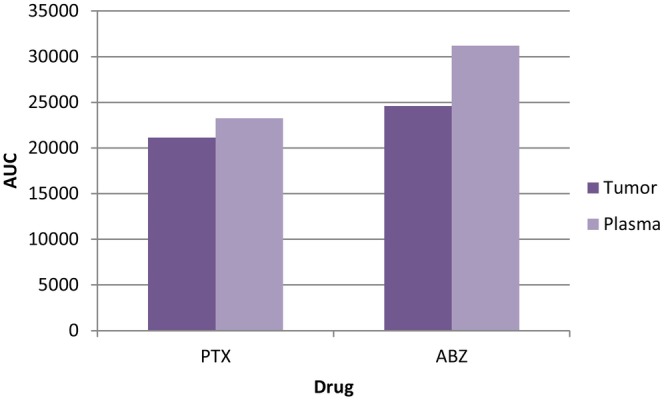
ABZ and PTX concentration in plasma and tumour at 3 h.

As per the graph shown in Figure 14, it showed that both drug albedazole and paclitaxel could be released from the APSTFM formulation and reached in the plasma and in tumour at 3 h.

## 4 DISCUSSION

The main goal of this endeavour was to develop targeted polymeric mixed micelles with ABZ‐PTX co‐delivery to boost therapeutic potential. Furthermore, co‐administration of ABZ‐PTX and TPGS‐FOL enhanced the target and synergistic action of polymeric mixed micelles. The drug organoleptic properties were observed and compared for its colour, odour, appearance and melting point. Compatibility between drug‐polymers was checked by using FTIR and results revealed the compatibility of drug and polymer with each other. DSC study showed purity of compound by its melting point detection. Micelles formation and its stability depend upon surfactant CMC value. TPGS showed 0.0205 mg/mL CMC values which indicated good dilution stability. TPGS‐FOL conjugation was prepared and confirmed by CMC and ^1^HNMR. The conjugation of TPGS with folate offers a multifaceted approach to targeted drug delivery, overcoming drug resistance, and improving the efficacy of anticancer therapies while minimizing systemic toxicity. This strategy holds great promise for the development of more effective cancer treatments. CMC value of TPGS 1000‐FOL was found to be 0.0015 mg/mL that showed better dilution stability of TPGS‐FOL than TPGS. Solvent evaporation method is used for preparation of polymeric mixed micelles. Drugs polymer ratios were optimized from preliminary studies and according to results obtained drug‐polymer combination was selected and effect of polymer ratios on mean size, PDI, zeta potential and %EE was evaluated. The drug ratio 1:1 (ABZ and PTX) was selected for further evaluation and factorial design study. Final optimized batch of formulation was estimated by 3^2^ factorial designs. According to the results, the polymer range was determined to be 100, 150 and 200 for soluplus and 220, 270 and 330 for TPGS. The effect of three levels (low, medium and high) and two factors that are polymer concentration were examined. Same batch composition applied for preparation of APSTFM. Evaluation of APSTFM micelles were performed for determination of drug loading, EE, mean size, zeta potential, PDI, FTIR, DSC, TEM, In‐vitro release, Ex‐vivo and In‐vivo investigation. The encapsulation of ABZ and PTX in APSTM was found to be 66.13% and 77.16%, respectively. The encapsulation of ABZ and PTX in APSTFM was found to be 57.13% and 74.81%, respectively. The size of the micelles did not appreciably alter after being diluted in water 5, 10 or 100 times. These findings revealed that APSTM and APSTFM were more resilient to dilution after injections. The observation of storage stability study revealed that micelles showed stability under 4°C for 45 days. So the storage condition of micelles should be at 4°C. The MTT assay results demonstrated that IC_50_ of APSTFM (0.2 ± 0.039 μg/mL) was much lower than free drugs ABZ (2 ± 0.015 μg/mL) and PTX (0.7 ± 0.039 μg/mL). So, higher cytotoxicity showed by APSTFM polymeric mixed micelles as compared to pure drug ABZ and PTX. Anti‐tumour activity was performed on nude mice and result showed that treatment with APSTFM strongly inhibited the growth of tumour during 15 days treatment. Pharmacokinetic study showed that both drug albedazole and paclitaxel could be released from the APSTFM formulation and reached in the plasma and in tumour at 3 h.

Nanomicelles loaded with dual drugs represent a significant advancement in drug delivery technology. The key novelties and advantages of proposed nanomicelles system over other systems are like it enhanced the solubility of poorly soluble drug, dual drug targeting, controlled release or sustained release properties and also it reduced toxicity. These features make dual drug‐loaded nanomicelles a promising approach in the field of drug delivery, offering potential improvements in therapeutic efficacy and patient outcomes over traditional delivery methods.

## 5 CONCLUSION

The study aimed at establishing the anticancer potential of ABZ and increased the therapeutic efficiency of the dual drug combination of ABZ and PTX. Further, ABZ PTX loaded TPGS‐Fol‐soluplus polymeric mixed micelles were successfully formulated and characterized. The compatibility of TPGS, folate ligands, and soluplus in the formulation ensures the stability and biocompatibility of the mixed micelles, making them suitable for systemic administration. Moreover, the inclusion of folate‐targeting ligands on the micellar surface facilitates active targeting of cancer cells, as FRs are often overexpressed on the surface of various cancer cell types.

The ABZ‐PTX‐Soluplus‐TPGS‐Fol micellar system outperformed conventional polymeric micelles due to its excellent entrapment efficiency and target specificity. The developed ABZ and PTX loaded soluplus & TPGS‐FOL conjugated polymeric micelles demonstrated smaller in size, good encapsulation effectiveness, greater stability upon dilution, sustained release, and biocompatibility. MTT assay and animal studies confirmed superior cytotoxicity of formulate micelles. The formulation of mixed micelles encapsulating two potent anti‐cancer drugs, albendazole (ABZ) and paclitaxel (PTX), utilizing a blend of TPGS, folate‐targeting ligand, and soluplus, represents a promising approach for cancer therapy. Thus, the study concluded that the nanosystem TPGS‐SOL‐FA polymeric mixed micelles assisted delivery of PTX and ABZ.

Further studies regarding complete release profile in animals, acute and chronic toxicity studies are a scope for further studies. In conclusion, the future aspects of the mixed micellar formulation encapsulating ABZ and PTX using TPGS, folate ligand and soluplus hold great promise for advancing cancer therapy. Through continued research and development efforts, this innovative formulation has the potential to improve treatment outcomes, minimize side effects, and offer new therapeutic options for cancer patients.

## AUTHOR CONTRIBUTIONS

**Nikita Maruti Gaikwad:** Conceptualization (equal); data curation (equal); formal analysis (equal); investigation (equal); methodology (equal); project administration (equal); resources (equal); software (equal); supervision (equal); validation (equal); visualization (equal); writing – original draft (equal); writing – review and editing (equal). **Pravin Digambar Chaudhari:** Conceptualization (equal); data curation (equal); formal analysis (equal); investigation (equal); methodology (equal); resources (equal); software (equal); validation (equal); visualization (equal); writing – original draft (equal). **Karimunnisa Sameer Shaikh:** Conceptualization (equal); data curation (equal); formal analysis (equal); investigation (equal); methodology (equal); resources (equal); software (equal); validation (equal); visualization (equal); writing – original draft (equal). **Somdatta Y. Chaudhari:** Investigation (equal); methodology (equal); project administration (equal); supervision (equal); visualization (equal); writing – review and editing (equal). **Sandeep S. Pathare:** Data curation (equal); formal analysis (equal); investigation (equal); project administration (equal); resources (equal); software (equal); visualization (equal); writing – original draft (equal); writing – review and editing (equal). **Amir Afzal Shaikh:** Data curation (equal); formal analysis (equal); investigation (equal); methodology (equal); software (equal); validation (equal); writing – original draft (equal); writing – review and editing (equal). **Nada H. Aljarba:** Data curation (equal); investigation (equal); project administration (equal); supervision (equal); validation (equal); visualization (equal); writing – original draft (equal); writing – review and editing (equal). **Ajoy Kumer:** Funding acquisition (equal); investigation (equal); project administration (equal); supervision (equal); validation (equal); writing – review and editing (equal). **Bikram Dhara:** Conceptualization (equal); funding acquisition (equal); project administration (equal); supervision (equal); validation (equal); visualization (equal); writing – review and editing (equal).

## CONFLICT OF INTEREST STATEMENT

All authors have no conflicts of interest to disclose.

## Data Availability

Data sharing is not applicable to this article as no new data were created or analyzed in this study.
